# A Treatise on Acute and Chronic Diseases of the Neck of the Uterus

**Published:** 1854-04

**Authors:** 


For sale by Miller, Orton & Mulligan. H.

---

ART. VI.—*A Treatise on Acute and Chronic Diseases of the Neck of the Uterus.* Illustrated with numerous Plates, colored and plain. By Chas. D. Meigs, M. D., Professor of Midwifery and Diseases of Women and Children in Jefferson Medical College, etc., etc. Philadelphia: Blanchard & Lea. 1854.

While we wrote the preceding notice of Bennett on the Uterus, this volume lay upon our table unopened. We have since given it a thorough and careful perusal.

Very many of our readers have already seen this essay, in that Bath-brick looking volume called the Transactions of the American Medical Association for 1853. We had noticed it there, and intended to have read it, but the number of volumes sent us from publishers, for reviews, deferred the task.

This monograph, as it now appears under the advantages of beautiful paper, large, clear type, and wide margins, is the neatest looking volume which has emanated from the American press for many years. It is a pity that its binding is not of that substantial character which would match so well with its internal beauties.

The illustrations (the same that appear in the Transactions of 1853) are, in some respects, fair specimens of colored lithography. They seem, however, to belong to the French school of art, in which artistic truth, and natural shading, are sacrificed to an Ethiopian fondness for gay colors. Such brilliancy of tint, here crimson, there green; now golden yellow, and then sky blue; with such charming backgrounds of body and fundus, glowing in all the colors of an Italian sunset, are not to be found in any ordinary squintings through the speculum. There is also some very "daring foreshortening," but on the whole they help not a little to illustrate the cases reported.

Come we now to the literary and professional character of the work.

The old leaven of transcendental phraseology still clings to Dr. Meigs. He has a propensity for writing his noun before his adjective, and for using unusual, or of coining unheard-of, words. Thus we have scattered in various localities through the volume the words "hypogaster," "sur-excited," "crispations," "spermzoon," "delimitary support," "iron revived by hydrogen." Most of these, and similar out-of-the-way words or phases, are intelligible from the context or derivation. In no place does it amount to unintelligibility, as it did in some of his previous works.

There is now scarcely more than enough of this Carlylish tinge to give an individuality to his style. It rolls off in a sort of *ore rotundo* utterance that makes the book very readable, inasmuch as it fixes the attention. For example, let us give this extract upon "The Ideal Uterus."

"And it is proper, indeed, that the physician should, in every case of disease, endeavor to acquire the perfect IDEA of the organ whose state he is about to determine. This determination he can only make, who hath already erected his ideal standard as now proposed—otherwise, he cannot but frequently err in his diagnosis. But he who hath ever at hand in his scientific store, a perfect IDEAL of the healthy organs, shall scarcely err, since, in every diagnostication, he will strictly compare the real with the perfect IDEAL or STANDARD, and, from observing the deviations and observations, deduce a perfect knowledge of the case before him."

Elsewhere he says that this ideal is made up of, in addition to a notion of its elements, one of its form, volume, place, posture in that place, sensibility, resistance, complexion, and all its powers as well as its anatomical relations or connections.

After perusing Dr. Meigs' really able vindication of the metroscope, its morality and necessity, we turned back to the title-page, expecting to find there, as the motto of the book, the single Latin word "*peccavi.*" We were disappointed, the more so when a friend at our elbow informed us of the struggles of modesty, and conscientious scruples, through which Dr. Meigs had arrived at the use of the now much lauded speculum. Philadelphia is proverbially slow in adopting improvements suggested by the outside barbarians; but its luminaries have one merit—when they find that they must yield, they do so in so graceful a manner that no one would dream they had ever pursued a different course.

Hence it is that the unsophisticated reader of this treatise acquires the notion that the speculum has ever been the pocket companion of the author. But the facts are, that in former times no one has so strenuously opposed its use as Dr. Meigs, and no one has more loudly advocated the necessity of sacrificing professional knowledge and success at the shrine of false modesty.

But this is comparatively unimportant. Dr. Meigs uses now all the appliances for obtaining knowledge within his reach; and he teaches intelligently what he knows. Dr. M. prefers to use the Recamier metroscope, and has a singular notion that it should be blackened within, to prevent chromatic rays of light, which might deceive the eye. But we do not make out from what he says that his own instrument is blackened, and opine that this is a mere bit of theory. Certainly the blackened tube would do away with chromatic rays, and, very probably, with all other rays.

We find but one essential difference in the views of Dr. Meigs from those of Bennett, and other writers who have preceded him. Dr. M. considers ulceration of the os or cervix uteri, as a very unusual occurrence, believing that the appearance of excrescences, mollusca, and fissures, so often observed, is due to growth or hypertrophy; that these parts are always covered with a thin pavement epithelium, which is often broken by awkward manipulation of the hand or metroscope.

As might be expected, the Nitras Argenti plays the principal character on the list of remedies. He speaks of the " destructive, indifferent, or antiphlogistic contacts of the nitrate of silver," and says that much skill is required in the use of the remedy. He uses principally the antiphlogistic contact, which is a medium between the indifferent and destructive contacts.

One thing we must not omit to mention. He holds that there is danger in *injecting* the uterus. It is, indeed, somewhat probable that the injection by a tube passed into the cervix is carried into the abdominal cavity by the Fallopian tubes.

The book begins and ends with a strenuous objection to making uterine diseases a specialty. Such authority as Dr. Meigs, speaking out for the integrity and unity of the profession, and that against his own personal interest, is worthy of attention, and should be commended to the advertising specialists in Boston and elsewhere. Dr. Meigs, who has given his talents and time for very many years to uterine disease, thinks that they can, or should be, best treated at home by the family physician; that they require no greater talent than any other inflammation for their treatment; and that the modest practitioner should not allow a few notorieties in great cities to monopolize a branch of practice to which he himself is fully competent.

